# Correction: B cell tolerance and antibody production to the celiac disease autoantigen transglutaminase 2

**DOI:** 10.1084/jem.2019086002062026c

**Published:** 2026-02-12

**Authors:** M. Fleur du Pré, Jana Blazevski, Alisa E. Dewan, Jorunn Stamnaes, Chakravarthi Kanduri, Geir Kjetil Sandve, Marie K. Johannesen, Christian B. Lindstad, Kathrin Hnida, Lars Fugger, Gerry Melino, Shuo-Wang Qiao, Ludvig M. Sollid

Vol. 217, No. 2 | https://doi.org/10.1084/jem.20190860 | November 14, 2019

The authors regret that, in the originally published version of their article, the IgL KI and IgH KI plots with TG2 tetramer staining in Fig. 1 C (middle images in bottom row) were accidentally duplicated from the IgL KI plot with anti-14E06 staining in the same figure (second image in top row). The original and corrected Fig. 1 are shown here. This correction does not change the original conclusions of the article, and the figure legend remains unchanged. The HTML and PDF versions of this article have been corrected. The error remains only in print and in PDFs downloaded before February 5, 2026.

**Figure:**
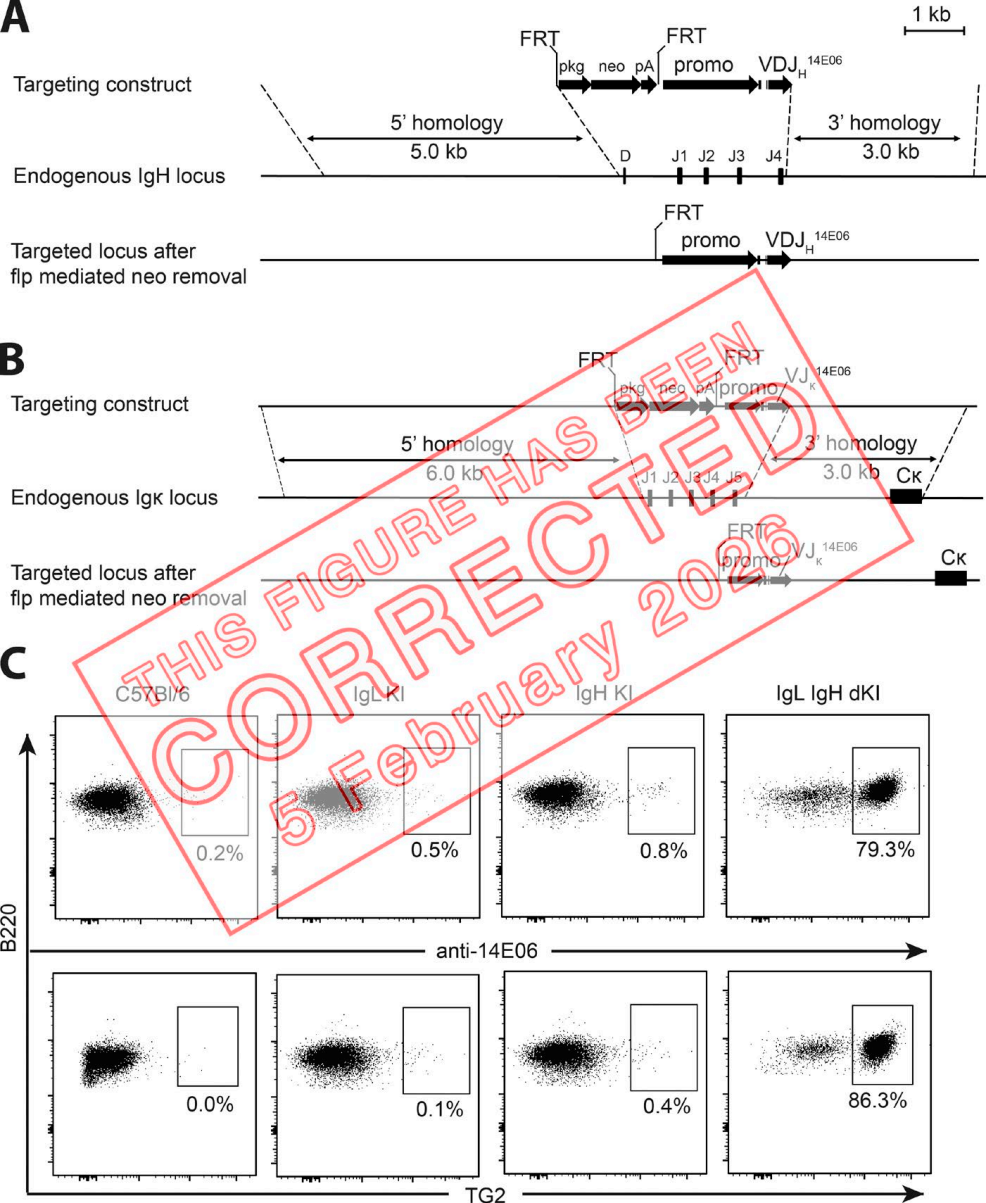


**Figure 1.**
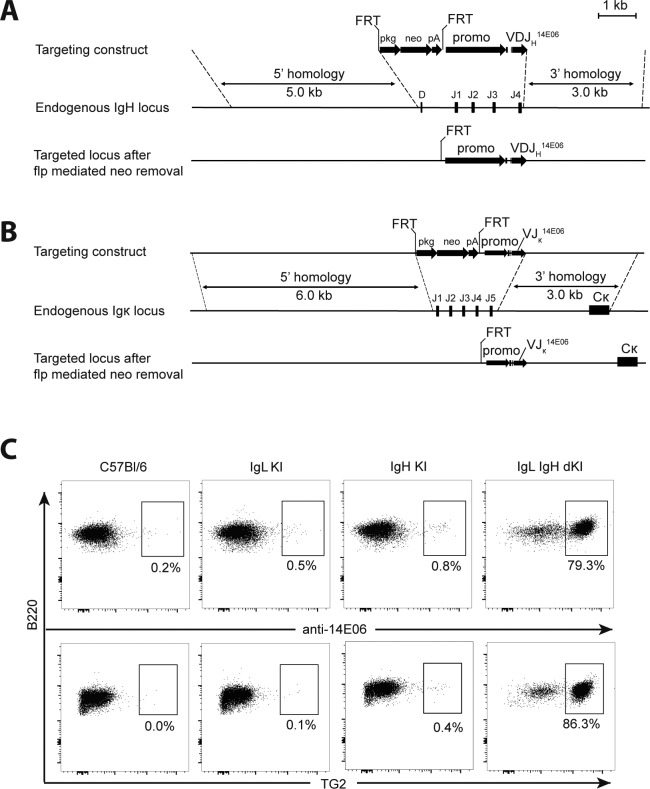
**Generation of 14E06 KI mice. (A)** Generation of the VDJH 14E06 KI mouse. Top: Targeting construct for the rearranged VDJH 14E06 and a FRT-flanked pkg-neo selection cassette. Middle: Mouse H chain locus with indication of the replaced D and J1-4 gene segments. Dashed lines indicate terminal points of homologous recombination. Bottom: Integrated transgene after flp mediated excision of the FRT-flanked neomycin selection gene. **(B)** Generation of the VJ_κ_ 14E06 KI mouse. Top: Targeting construct for the rearranged VJ_κ_ 14E06. Middle: Mouse κ L chain locus with indication of the replaced J1-5 gene segments. Bottom: integrated transgene after flp mediated excision of the FRT-flanked neomycin selection gene. **(C)** Detection of transgenic anti-TG2 BCR. Spleen cells from WT C57Bl/6 mice (obtained from Janvier Laboratoires), 14E06 L chain single KI and H chain single KI littermate control mice, and H and L chain double KI mice on C57Bl/6 background were stained using anti-B220 and an antibody specific for the H and L chain combination of 14E06 (anti-14E06) or mTG2 tetramers. Data are representative of two independent experiments.

